# Mortuary Statistics

**Published:** 1880-10

**Authors:** 


### AUGUST REPORT OF DEATHS AND INTERMENTS IN BALTIMORE.

| CAUSE OF DEATH. | No. | CAUSE OF DEATH. | No. | CAUSE OF DEATH. | No. |
|---|---|---|---|---|---|
| Angina Pectoris | 1 | Entero Colitis | 3 | Inflammation of Lungs | 1 |
| Apoplexy | 7 | " Phthisis | 1 | " Liver | 1 |
| Ascites | 3 | Embolism | 1 | " Pericardium | 1 |
| Asthma | 2 | Epilepsy | 2 | " Peritoneum | 3 |
| Asthenia | 7 | Effusion of Brain | 1 | Intemperance | 2 |
| Bright's Disease | 3 | Eczema | 3 | Jaundice | 1 |
| Burns | 2 | Exhaustion | 1 | Marasmus | 21 |
| Carbuncle | 1 | Fever, Bilious | 2 | Measles | 2 |
| Cancer, Bowels | 2 | " Brain | 1 | Meningitis | 10 |
| " Uterus | 2 | " Intermittent | 1 | " Tubercular | 2 |
| " Stomach | 2 | " Gastric | 1 | " Cerebro Spinal | 2 |
| Cancerous Cachexy | 1 | " Malarial | 2 | Old Age | 18 |
| Congestive Chill | 1 | " Remittent | 1 | Paralysis | 9 |
| Child Birth | 3 | " Scarlet | 31 | Pneumonia | 11 |
| Cholera Infantum | 65 | " Typhoid | 17 | " Typhoid | 1 |
| " Morbus | 6 | " Typho-Malarial | 7 | Premature Birth | 10 |
| Cirrhosis of Liver | 2 | Fracture Leg | 1 | Pyæmia | 1 |
| Congestion of Brain | 5 | Gangrene Senile | 2 | Rheumatism | 2 |
| " Liver | 1 | Gangrenous Stomatitis | 3 | Scrofula | 2 |
| " Lungs | 1 | Gastro Enteritis | 1 | Softening of Brain | 1 |
| Consumption of Lungs | 81 | " Intestinal Catarrh | 1 | Septicæmia | 2 |
| Convulsions | 27 | Gout | 1 | Stricture Œsophagus | 1 |
| Croup | 4 | General Paresis | 1 | Selerosis of Brain | 1 |
| Cyanosis | 4 | Hemorrhage, Lungs | 1 | Suicide | 1 |
| Difficult Parturition | 1 | " Post Portem | 1 | Syphilis | 1 |
| Dentition | 11 | " Umbilical | 1 | Tabes Mesenterica | 3 |
| Debility | 1 | Hydrocephalus | 5 | Tetanus | 3 |
| Diabetes | 1 | Hæmatemosis | 1 | Trismus Nascentium | 5 |
| Diarrhœa | 13 | Inanition | 17 | Toxæmin | 1 |
| Diphtheria | 24 | Indigestion | 3 | Ulcer of Leg | 1 |
| Disease of Heart | 16 | Injury of Spine | 1 | Uræmia | 1 |
| " Spine | 1 | Inflammation of Brain | 2 | Unknown, Infantile | 1 |
| Dropsy, (Gen'l) | 5 | " Bladder | 1 | Whooping Cough | 21 |
| " Abdominal | 1 | " Bowels | 10 | Wounds | 2 |
| Drowned | 7 | " Stomach | 2 | | |
| Dysentery | 4 | " Bronchi | 3 | Total | 596 |

THE DEATHS REGISTERED IN THE MONTH INCLUDE

| | | | |
|---|---|---|---|
| Under 1 year | 203 | Between 20 and 30 " | 44 |
| Between 1 and 2 years | 72 | " 30 and 40 " | 43 |
| " 2 and 5 years | 42 | " 40 and 50 " | 38 |
| | | " 50 and 60 " | 24 |
| Under 5 years | 317 | " 60 and 70 " | 30 |
| Between 5 and 10 years | 25 | " 70 and 80 " | 26 |
| " 10 and 15 " | 13 | " 80 and 90 " | 16 |
| " 15 and 20 " | 17 | " 90 and 100 " | 3 |

NATIVITY, ETC.

| | |
|---|---|
| United States—White | 373 |
| " Colored | 143 |
| Foreign | 80 |
| Total for the month | 596 |

Among the decedents were 46 above the age of 70 years, whose combined ages were 3,679 years, averaging 80 years.

Estimated Population, 393,796. White, 338,384. Colored, 55,412. Annual Death Rate during the month, whole population, per 1,000—18.15. White, 13,12. Colored, 31.20.

By Order: James A. Steuart, M. D., Commissioner of Health and Registrar.

A. R. Carter, Secretary.

U. S. Signal Office, Baltimore, Md., September 1, 1880.

### METEOROLOGICAL SUMMARY FOR THE MONTH OF AUGUST, 1880.

### PRESSURE.

| | | |
|---|---|---|
| Mean Monthly Barometer, | 30.070 inches. | |
| " " " for past 10 years, | 30.021 " | |
| Highest Barometer, | 30.432 " | on the 17th. |
| Lowest " | 29.745 " | on the 20th. |
| Monthly Range of Barometer, | .687 " | |

### TEMPERATURE.

Mean Monthly Thermometer, - - - - - 74.8 degrees.
" " " for past 10 years, - 76.0 "
Highest Thermometer, - - 91 degrees on 29th. Monthly Range, - - 30 degrees.
Lowest " - - - 61 " on 16th. Mean Daily Range, - - 15 "

### WIND, ETC.

Prevailing Direction of Wind, South.
Mean Velocity of Wind, 5.2 miles per hour.
Highest Velocity of Wind, 24 " " on 25th.
Total Movemeni of Wind, 3,876 miles.

Total Amount of Rainfall, 4.44 inches.
Number of Days on which Rain fell, 15.
Greatest Daily Rainfall, 1.35 inches on 25th.
Mean Relative Humidity, 70.6 per cent.

Robert Seyboth, in charge.

